# Qualitative inquiry with persons with obesity about weight management in primary care and referrals

**DOI:** 10.3389/fpubh.2023.1190443

**Published:** 2023-08-03

**Authors:** Lisa Bailey-Davis, Angela Marinilli Pinto, David J. Hanna, Michelle I. Cardel, Chad D. Rethorst, Kelsey Matta, Christopher D. Still, Gary D. Foster

**Affiliations:** ^1^Department of Population Health Sciences, Geisinger, Danville, PA, United States; ^2^Center for Obesity and Metabolic Research, Geisinger, Danville, PA, United States; ^3^Department of Psychology, CUNY Baruch College, New York, NY, United States; ^4^WW International, Inc., New York, NY, United States; ^5^Institute for Advancing Health through Agriculture, Texas A&M Agrilife Dallas Center, Dallas, TX, United States; ^6^Geisinger Commonwealth School of Medicine, Scranton, PA, United States; ^7^Perelman School of Medicine, University of Pennsylvania, Philadelphia, PA, United States

**Keywords:** referral and consultation, obesity, obesity management, qualitative research, delivery of health care

## Abstract

**Introduction:**

Referrals to evidence-based weight management in the community-commercial sector are aligned with clinical recommendations but underutilized.

**Methods:**

This qualitative study explored patients’ perceptions and expectations about obesity treatment in primary care and referral to community-commercial sector programs. Individual semi-structured interviews were conducted with a sample of US persons with obesity via telephone. Audiotape transcripts, interviewer notes, and independent review of data by two investigators allowed for data and investigator triangulation. Transcripts were analyzed using thematic analysis.

**Results:**

Data saturation was reached with 30 participants who had a mean age of 41.6 years (SD 9.4), 37% male, 20% Black/African American and 17% Hispanic, 57% college educated, and 50% were employed full-time. Three primary themes emerged: (1) frustration with weight management in primary care; (2) patients expect providers to be better informed of and offer treatment options; and (3) opportunities and challenges with referrals to community-commercial programs.

**Discussion:**

Patients expect that providers offer personalized treatment options and referrals to effective community-commercial programs are an acceptable option. If patient-level data are shared between clinical and community entities to facilitate referrals, then privacy and security issues need attention. Future research is needed to determine feasibility of implementing clinical to community-commercial referrals for obesity treatment in the United States.

## 1. Introduction

Persons with obesity (PwO) face personal, social, clinical, and health-system level challenges that converge in complex ways and function as barriers to effective treatment. To understand and manage these barriers, sequential steps in the treatment pathway have been outlined (1). The treatment pathway is assumed to begin by identifying oneself as overweight and followed by a desire to lose weight, weight loss attempt(s), seeking healthcare for obesity, and seeking care from a provider. A nationally representative cross-sectional study in the United States found that 92.7% of PwO perceive themselves as overweight, nearly all desire weight loss, and 6 in 10 have attempted weight loss (1). Few PwO (1 in 5) seek advice from a healthcare professional and among those that do, only 1 in 3 seek care from a provider (1). On average, PwO make 7 serious attempts at weight loss before seeking advice from a healthcare professional (2). Discordance between objective and perceived weight, desired and attempted weight loss, and weight-related stigma contributed to reluctance to seek help from healthcare professionals (1).

Once the topic of weight management is raised, providers are recommended to offer or refer PwO to intensive behavioral interventions but there has been little investigation of how PwO perceive the options and their involvement in deciding a course of action (3). Since effective weight management interventions include at least 12 sessions in the first year to address nutrition, physical activity, and behavioral skills (e.g., self-monitoring, problem solving, relapse prevention) (3) few providers are likely to have sufficient time or training in lifestyle medicine (4, 5) to offer them. Making referrals may be more feasible and will likely provide better outcomes (6). The opportunity to make a referral exists for providers within health systems but provider uptake may be poor, few patients may receive referrals and fewer may complete the referral. One in 5 providers in a large health system were responsible for over half of 17% of PwO receiving referrals to weight management (7). Among PwO who received a referral to a program within the health system, e.g., nutrition services, diabetes education, and bariatric surgical programs, 29% completed the referral (7). Improvements in providers making referrals and patients completing referrals are needed and, potentially, enhancing the variety of treatment programs will advance these improvements.

Commercial programs (WW, Jenny Craig, etc.) have been identified by nearly half of PwO and providers as effective weight management strategies (2). Regarding efficacy, PwO who received a referral to a commercial program lost double the amount of weight at 1 year compared to those who received provider advice (8). Notably, the rate of referral acceptance was 3 to 4 times higher (8) than that observed in US healthcare (7). While differences in out-of-pocket patient costs for clinical treatment (9) or commercial programs may influence referral acceptance, PwO did not identify finances as a leading barrier to initiating a weight management program (2). Importantly, participant household income was not detailed and many identified affordability of healthy food as a barrier to weight management (2). Despite acknowledgment that commercial programs are effective, it is unclear how these programs are perceived by PwO in the context of clinical care and the influence that provider, health system, or coverage would have on enrollment and participation.

Given the gaps identified, the purpose of this qualitative study was to explore the perceptions and expectations of PwO about discussing weight with providers, advice, and referrals. In the context of increased rates of obesity in the US and low implementation of clinical recommendations, a better understanding from the patient perspective about weight discussions, clinical advice, and referrals may advance public health goals to reduce obesity^1^ (2, 7, 10).

## 2. Methods

### 2.1. Participants

This study was conducted with US adults (≥18 years). A survey research company (Qualtrics) surveyed an existing panel of adults (convenience sample) to identify interest and eligibility based on a calculated body mass index (BMI) ≥30 kg/m^2^ (self-reported height and weight) and were English-speaking. Respondents were telephoned to achieve a purposive sample of approximately 25–30 PwO from urban, suburban, and rural communities, in total. Participant demographic data (age, race/ethnicity, sex, education, marital and employment status, weight perception) were collected by the interviewer. The study was approved by the Geisinger Institutional Review Board [2019-0292] and data were collected in winter 2019–2020.

### 2.2. Moderator’s guide

The moderator’s interview guide was developed by the study team and reviewed and enhanced by a patient advisory committee on obesity at [masked institution] (Supplementary Appendix A). The format included a moderator’s introduction, opening question, specific topics, and probes. The guide included questions about experiences and perceptions of adult weight loss, experiences discussing weight with providers, the nature of advice, and referrals for obesity treatment. Participant opinions about the potential value and challenges of receiving a referral from clinical to commercial weight management programs were explored.

### 2.3. Procedures

Qualtrics scheduled participant-researcher calls and an appointment reminder was emailed. Interviews opened by confirming the participant’s availability, receipt of study information and answering questions. All participants provided verbal informed consent. A semi-structured interview strategy was used to encourage conversation. Each interview was conducted by telephone, audio-recorded, and transcribed verbatim. All interviews were conducted by a trained qualitative interviewer (LBD). For their participation, interviewees received $50. Two research team members (LBD and AMP) read and discussed the transcripts as they were produced and reached consensus about the point of data saturation. The audiotape transcripts, the interviewer notes, and the independent review of two investigators allowed for data and investigator triangulation, respectively, which adds to the credibility and dependability of the interpretive findings (11).

### 2.4. Analysis

Data transcripts were the main unit of analysis, and each was read several times to obtain a sense of the whole and promote reliability in the results and interpretation. Transcripts were analyzed using thematic analysis, a process that involves six phases including familiarization with the data, generation of initial codes, searching for themes, reviewing themes, naming themes, and producing a final report (12). Researchers (LBD, AMP) used an open-coding approach to compare initial impressions (e.g., initial codes) from the data. After consensus was reached on initial codes, LBD applied an open-coding strategy to code all transcripts. Atlas.ti was used to manage the data. The coding output was evaluated, and discrepancies were discussed and resolved. Researchers derived the themes from the data using an inductive, constant comparative strategy through discussion of coded data to reach consensus on emergent themes.

## 3. Results

A total of 30 PwO participated out of 85 who were screened as eligible for participation. Participants did not dropout or refuse, *per se*, as data saturation was reached and there was not a need to continue to schedule interviews. Participants from rural/urban/suburban areas were equally distributed (Table 1). The mean age of participants was 41.6 years (SD 9.4), over a third were male, 20% Black/African American and 17% Hispanic, most had college degrees, and were working full-time. Most females were married whereas most males were single. Most participants perceived their weight as being overweight or very overweight although they used various terms to self-describe their weight status. Self-reported BMI was not made available to researchers after eligibility screening. Interviews lasted 21 min on average (range 16–28).

**Table 1:** Characteristics of persons with obesity interviewed.

		Males	Females	Total
		(*N* = 11)	(*N* = 19)	(*N* = 30)
Age (Mean, SD)		43.2 (5.9)	40.6 (11.2)	41.6 (9.4)
Education (*n*, %)				
	High School/GED	1 (9)	3 (16)	4 (13)
	Some College/Technical	2 (18)	7 (37)	9 (30)
	College	7 (64)	7 (37)	14 (47)
	Post-Graduate	1 (9)	2 (11)	3 (10)
Marital Status				
	Married/Living with Partner	5 (45)	10 (53)	15 (50)
	Separated	0 (0)	4 (21)	4 (13)
	Single	6 (55)	5 (26)	11 (37)
Employment Status				
	Full-time	9 (82)	6 (31)	15 (50)
	Part-time	0 (0)	3 (16)	3 (10)
	Unemployed	2 (18)	10 (53)	12 (40)
Weight Perception				
Very Overweight		2 (18)	5 (26)	7 (23)
	Overweight	8 (73)	14 (74)	22 (73)
About Right		1 (9)	0 (0)	1 (3)
Race/Ethnicity				
	Black, African American	1 (9)	5 (26)	6 (20)
	Asian, Pacific Islander	1 (9)	0 (0)	1 (3)
	White, Caucasian	6 (54)	11 (58)	17 (57)
	Hispanic	3 (27)		5 (17)
	Other	0 (0)	1 (5)	1 (3)
Community Type				
	Rural	1 (9)	9 (47)	10 (33)
	Suburban	5 (45)	5 (26)	10 (33)
	Urban	5 (45)	5 (26)	10 (33)

Three primary themes and several subthemes emerged from the inductive analysis (Table 2). Primary themes are: (1) frustration with weight management in primary care; (2) patients expect providers to be better informed of and offer treatment options; and (3) opportunities and challenges with referrals to community-commercial programs. Subthemes that emerged are identified and presented with primary themes below.

**Table 2:** Themes and subthemes from qualitative interviews with persons with obesity about weight discussions in primary care and referrals for treatment.

Themes	Subthemes
Frustration with weight management in primary care	
	When the focus is health, discussion is comfortable regardless of who initiates the topic
	Frustration with vague advice from providers
	Frustration with managing obesity on their own
Patients expect providers to be better informed of and offer treatment options	
	Expect information about how obesity interacts with other chronic disease management
	Expect treatment choices for a personalized approach
	Value provider support in decision-making
	Details needed to inform decision-making
	Expectations for maintenance and relapse
Opportunities and challenges with referrals to community-commercial programs	
	Referral arrangements
	Follow-up, accountability, and care coordination expectations
	Data privacy, security risks, and ethical challenges

### 3.1. Frustration with weight management in primary care

Participants were asked about their experiences with weight management discussions in primary care, who initiated the conversation, and the treatment received. Three subthemes emerged within the primary theme: (a) when the focus is health, discussion is comfortable regardless of who initiates the topic; (b) frustration with vague advice from providers; and (c) frustration with managing obesity on your own.

*When the focus is health, discussion is comfortable regardless of who initiates the topic*. Participants reported concerns about the impact of excess weight on physical, biological, and mental health. Weight-related comorbidities were reported including mobility limitations, back and knee pain, arthritis, prediabetes, type 2 diabetes, hypercholesterolemia, high blood pressure, breast cancer, sleep apnea, polycystic ovary syndrome, and depression. Prevention of comorbidities was a concern, and several indicated having a positive family history of obesity-related chronic disease. Participants reported being aware of health risks associated with excess weight given their life experience with obesity, a history of discussions with providers, and self-directed research.

“When I started the discussion, I told him about the research I’ve been doing online, and that changed the whole conversation into a very helpful conversation instead of an uncomfortable one.” -Female, age 36 years, post-graduate, full-time, married, overweight

In the context of health concerns, PwO initiated discussions about their weight or were open to the topic being raised by providers. Participants were ambivalent regarding concern of who raised the topic. Regardless of who initiated the discussion, provider attributes that contributed to comfortable conversations included empathy and a non-judgmental attitude.

“If the doctor doesn’t seem like they are attacking me with their comments about my weight and that they’re more trying to really look out for my health, that’s a lot more helpful to me, because I’ve had it go both ways and when it seems like a doctor genuinely cares about my health that’s what really matters to me.” -Female, age 30 years, some college, unemployed, living with partner, overweight

An established, respectful, and trusting relationship with the provider contributed to PwO comfort.

“I have a really good relationship with my primary care doctor, so she does a really good job, with just kind of letting me know what I need in a sort of up-front kind of manner. No sugar coating. I mean, I feel comfortable after having that discussion with her.” -Male, age 39 years, post-graduate, full-time, single, overweight

Conversely, PwO were uncomfortable when they felt labeled or judged by their weight status.

“Well, definitely nobody wants to be told they’re fat. Basically, my family doctor would just be, like, you are overweight. You need to lose weight, and it just was like kind of rude about it.” -Female, age 40 years, some college, unemployed, divorced, somewhat overweight

*Frustration with vague advice from providers*. Participants were frustrated by the content of conversations about weight as the advice was non-specific and behavioral recommendations were “always like a suggestion, it’s not like I am prescribing this to you.”

“Not specific. It was more of a conversation about moderation and then being physically active at least 30 min a day, but no set plan per se.” -Male, age 42 years, college, full-time, married, overweight

Participants reported that providers made false assumptions about their motivation, specifically that they were lazy or would not make a serious effort, and that this was a barrier to receiving useful advice.

“It just makes me feel like it’s just the same old thing, diet and exercise. You worry they assume you’re just lazy and you’re not so much, but with the physical pain it’s hard to exercise and so it just seems like circles kind of, the same old thing, same old thing. It’s like I can’t do a whole lot physically because I’m hurting. I need to get the weight off.” -Female, age 44 years, some college, unemployed, divorced, obese

Receipt of written materials about healthy eating, provider assistance in identifying gym memberships covered by insurance, and any follow-up appointment for weight management was uncommon. Some PwO reported receiving a referral from their primary care provider for weight management. Among those referred to a bariatric specialist, half had undergone a surgical procedure. Few PwO had been referred to a registered dietitian/nutritionist and others indicated a lack of awareness that a provider could make a referral to a bariatric specialist or dietitian. No participant had received an anti-obesity medication prescription, and few reported using over-the-counter supplements.

*Frustration with managing obesity on their own*. Participants reported feelings of being lost, confused, and frustrated with weight management care.

“…when you go to the doctor and weight loss is brought up, you almost feel as if they sent you out the door lost and confused and walking in circles with very minimal point in the right direction, so something like handing you a pamphlet, a booklet, a printout, anything with information…would be beneficial to a lot of people.” -Female, age 36 years, some college, part-time, living with partner, overweight

Participants described the efforts to manage their weight as being self-directed and independent of their provider. PwO reported being left on their own to find an effective approach, yet they would welcome provider involvement.

“Unfortunately, I don’t always think of my doctor when it comes to weight loss. I don’t feel like they don’t always look for that. I feel like people are kind of left on their own sometimes to figure that out. So, it would be nice if it was woven into a medical visit.” -Female, age 45 years, post-graduate, part-time, married, overweight

The experiences associated with frustration were related to primary care.

“You know, it just makes you wonder just why it took my orthopedic doctor to do this and he’s not really even into that kind of, it should have been my primary care that did it [made referral to bariatric program] a long time ago.” -Female, age 44 years, some college, unemployed, divorced, obese

In contrast, PwO who discussed their weight with a specialist (obstetrics/gynecology, orthopedics, endocrinology, bariatrics) described the advice as useful.

“She has given me pamphlets and some suggestive eating, diet things. Like for breakfast, different things that are good for diabetes and weight management, *Choose My Plate*, …things you can follow every day that will lead up to eating healthier and losing weight, getting control of my weight … no requirements of coming back to discuss it with her.” -Female, age 57 years, college, unemployed, single, obese

### 3.2. Patients expect providers to be better informed of and offer treatment options

In this second primary theme, five subthemes emerged and include: (a) expect information about how obesity interacts with other chronic disease management; (b) expect treatment choices for a personalized approach; (c) value provider support in decision-making; (d) details needed to inform decision-making; and (e) expectations for maintenance and relapse.

*Expect information about how obesity interacts with other chronic disease management*. Participants expect more information from providers about how their physical, biological, and mental health conditions interact with obesity. Participants voiced concern about the complexity of their conditions and their expectation to learn more about how obesity management could affect their cardiometabolic conditions, cancer remission, pain, and mental/emotional well-being.

“I would expect them to talk about what benefit that’s going to have on me managing my past conditions and preventing any future conditions, maybe alleviating any pain that I have like back pain or leg pain and things. I would also expect, because of the history of breast cancer, them to talk about that, like what effects that would have on any recurrences or things like that.” -Female, age 36 years, some college, unemployed, single, overweight

Conversely, some PwO were concerned about the side effects of their prescribed medications for comorbidities on weight management.

“I have bipolar disorder, anxiety disorder, and PTSD and so some of the medicines that I take do increase a little bit of my weight.” -Male, aged 47 years, some college, unemployed, single, heavy

Participants expect that providers will provide pathophysiological insight about obesity that helps them to better understand their situation and the benefits of weight management.

*Expect treatment choices for a personalized approach*. Participants expect providers to present treatment choices that are personalized. Specific personal factors that they expect to be considered are comorbidities, age, and sex.

“I would expect them to deliver it to me as personalized as can be. I wouldn’t feel comfortable with just a common program that has worked for some people that are not in the same situation as me, so I would appreciate it if it was kind of personalized and understood that this may help for my situation.” -Male, age 33 years, college education, full-time, married, overweight

Additionally, PwO expect details about program effectiveness (weight outcomes, sustainability of weight loss) related to these factors.

“I would like to know the success rate of people that are similar to me.” -Female, age 57 years, GED, disabled, separated, overweight

Some expect the provider to understand their prior history of weight management attempts, successes, and challenges to inform treatment choices.

“I would want more specifics, remember they went to school for that, they’re more informed than I am.” -Male, age 54 years, college, full-time, married, overweight

Balanced with a strong interest in a personalized approach, PwO expect the provider to make treatment recommendation(s), and they maintain an expectation of making a choice.

“Multiple options, that way the choice would be mine to make.” -Female, age 49 years, some college, retired, married, overweight

*Value provider support in decision-making*. Despite the general frustration with provider advice about weight management, PwO respect provider input and highly value their support in making a treatment decision. Participants anticipated that a provider’s recommendation would help them feel comfortable in making a decision.

“I think if my doctor were to say, listen, I’m aware of what your struggles are and I’m also a believer in this program, I think this could work. This is a good match. That would make me feel more comfortable, and also allow me the opportunity to discuss my experience with the doctor.” -Female, age 45 years, post-graduate, part-time, married, overweight

“It makes me feel that they trust and feel that the company is good and what they have to offer could benefit me. I value my doctor’s opinion.” -Female, age 44 years, some college, unemployed, divorced, obese

If the treatment choice was a referral to a program, the provider’s recommendation could help the PwO focus their research on trusted and effective programs.

“There’s a chance I may or may not follow through with it. If [the doctor] recommended it, I’m more apt to follow through and stick with it, because chances are he’s just looking out for my benefit.” -Male, age 40 years, GED, unemployed, single, very overweight

“It feels more genuine, it just feels like if I’m having a health care professional give me this information, it just feels more real and respectable then just something that I just happen to come across while browsing online.” -Female, age 34 years, some college, full-time, married, overweight

Many PwO anticipated taking additional steps to learn about programs that were provider-recommended before making a treatment decision.

“Their recommendation added to research online would lead me to find out what would be best for me. At first, I would probably try their advice.” -Male, age 43 years, college, full-time, single, overweight

“I would prefer for her to make a recommendation because I really trust my primary care doctor, so I would want her to make a recommendation and then I would do my research on my own.” -Male, age 39 years, post-graduate, full-time, single, overweight

*Details needed to inform decision-making*. Participants are concerned about treatment costs and insurance coverage. Regardless of whether PwO felt they had the resources to pay out-of-pocket for treatment, this issue was top of mind.

“I would expect probably a little bit of a run down on, there are so many out there right now, whether you’re cutting carbs or you’re cutting fat or calories. I’d probably want to know what style that was to see if it was even something that would interest me in my likes of food. The success rate would be nice. I know it’s not always easy to have those statistics, but that would be nice; and maybe even some pamphlets just like little information about what this program has to offer. Maybe whether it’s an exercise regimen or daily meal plan or you know whether they have a protein drink line. Things like that. Yeah, that would be helpful from the doctor.” -Female, age 30 years, some college, unemployed, living with partner, overweight

Participants appreciate the nuances of insurance coverage and plans and do not expect their providers to be able to fully represent costs other than a general range.

“Well, being a penny pincher, if [provider] had presented [weight management program] to me in a way where I felt like I had to do it, then I would have done it, of course. With programs and all these other things medical-related and insurance, I’m pretty skeptical and also pretty dissatisfied with the way insurance works … The trouble is asking how much classes were, and he didn’t know. He told me to ask somebody else, and somebody else told me to ask somebody else, and it was just a circle of cycles … Everybody has different insurance and maybe it’s hard for them to communicate it, or a company to communicate it, but I would think they could still communicate a range at least or something” -Male, age 42 years, post-graduate, full-time, married, slightly overweight

Treatment details such as nutrition modifications, physical activities, program format (virtual, in-person, hybrid; group, individual), schedule (synchronous/asynchronous, frequency, session length, program duration), safety, location, effectiveness, and weight loss sustainability are among the details that participants need to inform treatment decisions.

“…if I could find something that I could go like once, maybe twice a month to an actual physical location and do the rest of it online, that would be a major convenience.” -Male, age 40 years, GED, unemployed, single, very overweight

“That it’s healthy. It’s safe. That it is a good alternative to trying to do it on your own. It’s the best thing for my case specifically.” -Female, age 40 years, some college, unemployed, divorced, somewhat overweight

“Are the results only while the program is taking place or are they lasting?” -Male, age 42 years, college, full-time, married, overweight.

Details could be communicated verbally, with written materials, or by directing PwO to a website, perhaps noted on a coupon or voucher.

“…I wouldn’t expect the doctor to provide me with all the details because it is not their specialty, but maybe just a brief summary of what it’s all about … at least give me some sort of collateral like a pamphlet that would describe the options.” -Male, age 33 years, college education, full-time, married, overweight

*Expectations for maintenance and relapse*. Participants acknowledged the chronic nature of obesity and expect care to continue after weight loss is achieved.

“I would hope it would be open forever. The weight loss and the weight maintenance thing to me is an ongoing battle. It is not like some curable disease that once you are done with it, you don’t have to worry about it.” -Male, age 43 years, college, full-time, single, overweight

However, PwO had two perspectives regarding whether weight management should be a covered benefit after an initial successful weight loss (interviewer suggested 5%–10% but many participants identified 20% as successful weight loss). Some PwO expect weight management and maintenance to be a continuously covered benefit whereas others expected this to be limited to an initial attempt.

“Well, yeah, I was thinking well that would be my fault, but I guess that's what I paid for. I guess if I broke my leg and then 2–5 years later, I broke my leg again, I would still expect them to pay for it.” -Female, age 36 years, some college, unemployed, single, overweight

Those that held the latter opinion anticipated self-blame for weight regain and would not expect a payor to cover subsequent attempts.

“I would think it wouldn’t be [covered] if it was after 2 years, but I mean it is always a possibility, and it would be my own carelessness, so they helped me once, they may not help me again type of thing.” -Female, age 30 years, some college, unemployed, living with partner, overweight

### 3.3. Opportunities and challenges with referrals to community-commercial programs

Participants conceptualized the possibility of their provider offering a referral for weight management to programs including WW (formerly Weight Watchers, *n* = 29), gyms (*n* = 21), NutriSystem (*n* = 16), Jenny Craig (*n* = 8), Curves (*n* = 6), and Noom (*n* = 1). Three subthemes emerged related to the potential value and challenges of receiving a referral from a provider to a commercial weight management program, primarily focused on WW, including: (a) referral arrangements; (b) follow-up, accountability, and care coordination expectations; and (c) data privacy, security risks, and ethical challenges.

*Referral arrangements*. All but one participant was enthusiastic about the potential to receive a referral from their provider to a commercial weight management program. The participant that did not endorse a referral noted a preference to navigate programs independently. Many indicated that receiving a paper coupon or digital code would be acceptable.

“…it feels like [a referral from a primary care provider] would be a good motivator to check those things out and then start with a coupon, a code, for a discount of some kind, that makes it even more motivating so, yes, I would be interested.” -Female, age 34 years, some college, full-time, married, overweight

In this workflow, a PwO could research the program after discussion with the provider, navigate cost and coverage with their insurance company or the program directly, and decide to sign-up with a code or coupon. Several PwO described the coupon or code as a valued incentive or a “push” to act.

“Well, doing it on your own is kind of an elective choice. Doctor giving you recommendations on fixing the issue probably makes it more really … an issue … [a referral from a primary care provider] might give some people more comfort. Honestly, like okay, let’s do this. Well, it’s like more confidence, maybe it would be the push that they needed to get started on a healthier lifestyle, healthier path.” -Female, age 23 years, less than high school, unemployed, married, overweight

Some PwO indicated a preference to pick up a coupon in a waiting room rather than direct receipt from a provider. Other PwO indicated a preference for the provider’s office to manage a referral to a commercial weight management program in a manner like the existing workflow for specialist referrals.

“It feels like they put [traditional referral within health system] back on me. I would have preferred that they had taken the reigns and set something up, but my recollection is they just suggested a couple of things and it was on me to determine that these other people were in network or wherever, and all that.” -Male, age 42 years, post-graduate, full-time, married, slightly overweight

*Follow-up, accountability, and care coordination expectations*. Participants anticipate that a referral implies a partnership in pursuing weight management. As such, PwO see themselves as accountable to the provider in that they agree to the referral, will start the program, and will participate in the weight loss attempt.

“Overall, I think it would help being held accountable through a program, and I’m more of a person that everyone has to be on a system, planned out. If I have a schedule to follow, actually I will hold myself more accountable. Also, if I am seeing doctors, I just feel like it would definitely help me, push me to where I need to be. Then ultimately help with my overall health.” -Female, age 40 years, some college, unemployed, divorced, somewhat overweight

In turn, providers are expected to follow-up with the PwO to evaluate progress, suggest changes in treatment, and make modifications to their care plan.

“Hopefully, the doctor would want to check in with me, and say, alright, what did you do? Were you doing in-person interviews, were you doing it online? I think I would want to know that I have that communication available to me.” -Female, age 45 years, post-graduate, part-time, married, overweight

“Maybe add a medication, change something, change the doses, take a medication or two off my list.” -Male, age 40 years, GED, unemployed, single, very overweight

Participants envision that the partnership model includes the commercial weight management program and that the program could share the participant’s progress with the referring provider to facilitate care coordination.

“Yes, because I would consider it a partnership if he made that suggestion and I’m following that program, then I would expect him to have vested interest in how I’m doing.” -Female, age 34 years, college, full-time, married, overweight

“If they’re sharing the information, I would expect him to evaluate my progress or no progress, whichever case it is. If they are sharing everything. Whatever it is they tell you, I would expect them to follow up on that and talk to me about it. If anyone of them sees any sort of progress I would expect someone to contact me with the information. If they are doing something for my benefit, I believe the more information they have or they share, it’s going to be in my best interest instead of hiding anything between them.” -Male, age 54 years, college, full-time, married, overweight

Participants anticipate that providers, or their designee, would call, email, or text them about changes to medication when weight loss is achieved. Some PwO anticipate that the provider would routinely address progress, maintenance, and relapse during care visits.

“Oh, I think that would be fantastic because that’s saying that it’s not just a one and done, but he really believes in the change that needs to take place in my own life … if you are following up in 3 months, 6 months, whatever, there is an accountability that is there, and it’s also a piece that allows him to better assist and serve my needs.”-Male, age 42 years, college, full-time, married, overweight

“That would actually show that they care a lot and they are not just there for the paycheck, just shipping you off to somebody else.” -Female, age 23 years, less than high school, unemployed, married, overweight

*Data privacy, security risks, and ethical challenges*. The concept of data sharing directly between the provider’s office and a commercial program, regardless of whether this was for arranging a referral or coordinating care, raised concerns about data security and privacy for some PwO. Clearly written disclosures and limited data sets (weight, height, obesity comorbidities but not social security number) were mentioned as strategies to address these concerns.

“It would be good to have a disclosure or something that limits [commercial weight management program] as to what they can have specifically.” -Female, age 65 years, college, full-time, married, a little overweight

“As far as my weight and height, I don’t care, but if it has my social security on it, that would concern me. But just basic information about my height, weight, age, I don’t think that would be a problem for me.” -Female, age 45 years, post-graduate, part-time, married, overweight

Participants perceive that their health data are protected and secure in the health sector but have the potential to be “‘hacked” or “sold” in the commercial sector.

“I think the doctor’s office probably has extensive protection on their computers … I don’t know how well [commercial weight management program] would.” -Female, age 44 years, some college, unemployed, divorced, obese

“I am worried about my information getting sold to everybody and their brother” -Male, age 40 years, GED, unemployed, single, very overweight

Other PwO were ambivalent noting that much information is publicly available and shared in social media. Participants raised concern about provider and program integrity if a financial relationship was suspected in that the provider receives payment for program referrals. Participants speculated that such a relationship could lead to biased program referrals that may not be in the patient’s best interest.

“I would prefer a choice, that way I wouldn’t think that there was some type of kickback or referral system in place.” -Male, age 50 years, college, full-time, overweight

## 4. Discussion

These findings give voice to PwO regarding how providers can engage them in discussions about weight management, their expectations for advice about treatment choices, how to involve them in decision making, and the potential to refer PwO to commercial weight management programs. Our findings suggest that PwO are not seeking treatment from healthcare providers because the quality of care they have received has been poor and leaves them frustrated. PwO may also experience weight-related social stigma from providers and therefore delay in seeking treatment (13). This stigma further isolates patients and reinforces self-management. PwO therefore feel they lack guidance for their weight loss approaches due to a lack of information regarding impactful lifestyle changes, medications, and surgical options that may help them. Treatment care gaps identified in nationally representative cross-sectional studies reemerged in this qualitative inquiry (1, 2). Consistent themes were the identification of having obesity, a desire to lose weight, multiple attempts to lose weight, limited advice-seeking from a healthcare provider, and obesity self-management independent of medical oversight.

Consistent with expert recommendations on patient-centered care (14), PwO expect productive and meaningful patient-provider discussions about weight management, advice, and arrangement of referrals for obesity. While providers deprioritize addressing obesity, patients would highly value provider input. PwO are willing to have discussions on obesity if providers approach the topic professionally and with effective advice. Interactions with providers are hoped to be compassionate and informative about their condition. PwO have a strong desire to be involved and informed about their obesity and how it interacts with their comorbidities. PwO also want providers to be competent enough with obesity management to give varied options or to offer a personalized plan. Our findings are consistent with an Australian qualitative study investigating patient expectations related to chronic disease management that found patients expected health outcomes to result from patient-provider interactions, available treatment options, and their own actions (15). Although obesity is considered a medical disease, it is not often treated as one, leaving patients to address it themselves.

Robust interest in referrals for obesity treatment in the community-commercial sector, outside the traditional US healthcare system, is a unique finding that is perhaps explained by PwO self-management due to limited care and referrals. An Australian study observed that patients distinguished between individual provider agency and the healthcare system in treating chronic disease, whereas our findings suggest that PwO expect providers in the US to be competent and to offer referrals to evidence-based programs (15). PwO offered critique about providers, but less about the healthcare system, *per se*, and instead offered insight to potential system solutions. As stated, PwO experienced vague advice, non-routine follow-up, and few referrals. Regarding competency, providers receive minimal training and education in obesity management (4, 5). Despite the USPSTF standard of care for obesity being the provision of behavioral lifestyle advice and follow-up care in the office or making referrals, few providers do this and do it well (3, 4). Regarding non-routine follow-up, cross-sectional research found that only 24% of PwO had a scheduled follow-up to initial weight-related conversations (2). In addition, structural barriers, like time constraints on appointments, can manifest in the clinical decision to deprioritize time-intensive behavioral lifestyle counseling.

Referrals may be a promising solution given the conceptual, structural, and cultural barriers, specifically weight stigma, that interfere with providers’ communication of behavioral treatment strategies (16–18). PwO endorsed the idea of receiving direct referrals from their provider to effective obesity treatment programs in the community-commercial sector, a strategy with demonstrated feasibility and effectiveness in United Kingdom but not yet available in the US (7, 8). Advice given in a clinical appointment could then be implemented by programs in the community-commercial sector, especially if data regarding the PwO’s care plan could be shared between these two entities. Privacy and security concerns would need to be addressed as well as the communication flow, primarily to ensure that the PwO has consented to receive the referral. This solution could dually address the provider’s dwindling time with the PwO and shortfalls in provider competency, ensuring that personalized care with focused time for behavioral counseling in weight management could be delivered by trained and competent professionals. Patient education about how to choose an effective obesity treatment program may be warranted and paired with clinical communication to support shared decision making and referrals that will result in participation.

Treatment and maintenance costs were pervasive concerns, as they are with other chronic diseases, but PwO prioritize the need for system changes to better treat obesity. Presently, there are opportunities to meet their needs. The USPSTF concluded with moderate certainty that offering or referring PwO to intensive, multicomponent behavioral interventions would have a moderate net benefit (B recommendation) (3). Under the Affordable Care Act, most employer-sponsored insurance plans are required to cover, without cost-sharing, evidence-based USPSTF recommended preventive services that have a rating of A or B (19). Coverage for preventive services without cost-sharing applies only to Medicaid expansion and others enrolled in Alternative Benefit Plans (19). Coverage under Medicare Part B is more limited but provider-delivered weight-management is covered for PwO (19). Conservatively, if 35% of US adults have obesity (3) and more than 20 million adults are covered by private or Medicaid expansion (19), then 7 million PwO may be able to access weight management interventions, if available. The community-commercial sector could play a critical role in increasing the availability of interventions and promote referrals from the healthcare sector.

The strength of this study is insights from PwO regarding their experiences in clinical care, expectations, and opportunities for enhanced treatment outside of the traditional healthcare system. The study included a US sample with voices in rural, urban, and suburban areas that self-identified as having overweight or obesity. Qualitative methodologies offer insight into experiences and perceptions, but the findings do not represent, nor are they generalizable to, a broader and larger cross-section of the US population. Additionally, qualitative data interpretation is subject to bias, though triangulation strategies were employed to minimize bias.

Obesity is known to be a chronic medical condition yet is not routinely addressed by providers. PwO identified perceived roadblocks to care that are mainly based on non-specific advice, limited information about treatment options and follow-up care, and few referrals. Effective community-commercial programs as a referral option is an acceptable concept to address this care gap and may advance progress in achieving public health goals but research is needed to determine feasibility in the US healthcare system.

## Data availability statement

The raw data supporting the conclusions of this article will be made available by the authors, without undue reservation.

## Ethics statement

The study was approved by the Geisinger Institutional Review Board [2019-0292]. Written informed consent for participation was not required for this study in accordance with the national legislation and the institutional requirements.

## Author contributions

LB-D, AP, CR, CS, and GF contributed to the study conception and design. Material preparation, data collection, and analysis were performed by LB-D, AP, and DH. The first draft of the manuscript was written by LB-D. LB-D, AP, DH, MC, CR, KM, CS, and GF commented on the previous versions of the manuscript. All authors contributed to the article and approved the submitted version.

## Funding

This study was funded by a grant from WW International, Inc.

## Conflict of interest

LB-D and CS received a research grant from WW International, Inc. AP was employed by WW International, Inc. during the design phase. CR was employed by WW International, Inc. during the conduct of the study. GF and MC are employed by and are shareholders of WW International, Inc.

The remaining authors declare that the research was conducted in the absence of any commercial or financial relationships that could be construed as a potential conflict of interest.

## Publisher’s note

All claims expressed in this article are solely those of the authors and do not necessarily represent those of their affiliated organizations, or those of the publisher, the editors and the reviewers. Any product that may be evaluated in this article, or claim that may be made by its manufacturer, is not guaranteed or endorsed by the publisher.

## Footnotes

^1^ https://health.gov/healthypeople
