# Supplementary material for: Qualitative inquiry with persons with obesity about weight management in primary care and referrals
Source: Front Public Health. 2023 Aug 3;11:1190443. doi: 10.3389/fpubh.2023.1190443 (PMC10435859; doi:10.3389/fpubh.2023.1190443)
Supplement: Supplementary file 1 [file Table_3.DOCX]

**Supplementary Material**

**Appendix A. Interview Questions**

1. Tell me a little about yourself:
2. Highest level of education: HS/GED, BS, Graduate
3. Employment status: FT, PT, unemployed
4. Age: years
5. Sex: M, F, non-binary
6. Marital status: Married, separated, single, widowed
7. Perception of current weight status: very overweight, overweight, about right
8. When was the last time that you talked about weight loss with a doctor?
   1. What type of doctor(s) have ever discussed weight management with you? (Probe: primary care, cardiologist, OB/GYN, etc.)
   2. Thinking about the most recent time, who initated the conversation about weight, you or the doctor(s)?
      1. Is it helpful when you bring it up versus your doctor?
      2. Did your doctor ask if it was okay to discuss your weight?
   3. What was the major reason why weight loss was discussed in the doctor’s office? (Probe: health, blood pressure, labs, mobility)
      1. Has a doctor ever discussed how weight loss could prevent or improve diabetes control?
      2. Has a doctor ever discussed how weight loss could help manage high blood pressure, heart disease, orthopedic issues, fertility, or prevent certain cancers?
   4. How did you feel about how your weight was discussed?
      1. What, if anything, helped you feel comfortable with the discussion?
      2. What, if anything, made you feel uncomfortable?
9. Weight loss guidelines recommend diet, physical activity, medications, and/or surgery.
   1. Which of these have you tried on your own, without ever discussing with a doctor?
   2. Which of these has a doctor ever offered you in his/her office or referred you?
      1. Did the doctor ask about your preference?
      2. Why did you accept some referrals but not others? (Probe: trust referring provider, doctor recommended, believe will be effective, convenience, cost)
      3. To what degree did you follow the doctor’s advice or program?
      4. How satisfied were you with the result?
   3. If you were refered to care outside of the doctor’s office, how was the referral arranged- by the provider, someone else in the office, insurance company, or did the program contact you directly?
      1. How satisfied were you with the referral process? What would you change?

1. If your doctor referred you to a weight loss program, what would you expect him/her to say about the program? (Probe: effectiveness, cost, convenience, safety)
   1. Would you expect that the provider discuss a few programs and let you make the choice or that he/she makes a recommendation about a specific program?
      1. Would you like a few options or one specific program that the doctor believes in for everyone?
      2. How important is it to you to know how a specific program works for someone like you, for example, similar age or lifestyle or facing similar health conditions?
   2. What are the top three community-based or online commercial programs that you might expect as potential referral options? (Probe: WW, Weight Watchers Reimagined, if not mentioned)
   3. What details would you need to follow through with starting the program? (Probe: written materials, website, toll-free number, insurance coverage, cost, format- group or one-on-one, in-person/telephone/online)
2. Referrals could be distributed by doctors via a coupon with a website address and a discount code. This could print with an after visit summary when you check out. You could use this code to choose a WW program that fits your preference (e.g., digital, in-person, both).
   1. How likely would you be to use the coupon code?
   2. In your opinion, what is the added value for you, in receiving a referral to WW from a doctor vs doing it on own?
   3. What concerns and opportunities do you envision with this process?
   4. Would you be okay with the doctor checking in with you directly about your

progress? How should the doctor follow-up with you- office visit, telephone call, email, text? How often?

- 1. If you provided consent to share certain data like weight history and related health conditions, what concerns would you have about your doctor’s office sharing with WW? What concerns do you have about doctor and WW communicating directly about your progress?
     1. What benefit might this offer to you? (Probe: convenience, cost, coordination)
     2. What risks could this present for you? (Probe: lack of confidentiality, scope creep, insurance concern)
  2. What, if any, information would you want WW to share about you with your doctor? (Probe: attended a session, signed up for a program, weight loss, weight measures)
     1. What information would you not want them to share?
  3. How would you want your doctor to use this information? (Probe: positive progress vs. lack of progress)
  4. Highly engaged- After you lose 10% or reach your goal weight, what do you expect to happen next? (Probe: Maintenance) what if there is a relapse, say 2 years later and you regain weight-should you be allowed to restart the intensive program? If insurance paid the first time, should insurance pay a second time?
